# Plastome evidence repositions *Abelmoschus ficulneus* as a close relative of cultivated okra (*Abelmoschus esculentus*, Malvaceae)

**DOI:** 10.3389/fpls.2026.1877509

**Published:** 2026-07-22

**Authors:** Mohamed Hassan Korkar, Hagar Tarek Elhefnawi, Yosur Gamal Fiteha, Samah Mohamed Rizk, Abeer A. Faiesal, Mohamed Abdel-Salam Rashed, Mahmoud Magdy

**Affiliations:** 1 Genetics Department, Faculty of Agriculture, Ain Shams University, Cairo, Egypt; 2 Basic and Applied Agricultural Sciences Department, Higher Institute for Agricultural Cooperation, Cairo, Egypt; 3 Plant Biology Department, Faculty of Biology, Murcia University, Murcia, Spain

**Keywords:** chloroplast genome, codon usage bias, dN/dS ratio, hypervariable loci, IR boundary dynamics, molecular markers, nucleotide diversity, simple sequence repeats

## Abstract

**Introduction:**

The genus *Abelmoschus* (Malvaceae) includes cultivated okra (*A. esculentus*), yet its phylogenetic relationships remain unresolved using limited molecular markers.

**Methods:**

In this study, complete chloroplast genomes of *A. esculentus* and the first complete plastome of *A. ficulneus* were assembled and analyzed together with published plastomes of three additional *Abelmoschus* species and *Hibiscus* rosasinensis as an outgroup

**Results and Discussion:**

Plastome sizes ranged from 163, 119 to 163, 503 bp, with conserved quadripartite structures and GC content (~36.7%). A total of 751 and 755 simple sequence repeats (SSRs) were identified in AE12 and AF01, respectively, with hexanucleotide repeats as the dominant class (~25%) and a consistent enrichment in the LSC region (~62–64%). Codon usage analysis of 64 codons revealed a conserved bias toward A/U-ending codons, with no substantial interspecific variation. IR boundary analysis identified a genus-specific feature, with rps3 consistently located within IR regions in *Abelmoschus*, in contrast to *rpl2* in *Hibiscus*. Polymorphism analysis across 271 genomic regions identified nine hypervariable loci, all exhibiting five haplotypes (Hap = 5) and maximum haplotype diversity (Hd = 1). Nucleotide diversity was low overall (mean p = 0.00116). Phylogenetic reconstruction based on complete plastomes resolved two strongly supported clades (SH-like ≥ 0.99) and consistently placed *A. ficulneus* with *A. esculentus*. Hypervariable loci including *ycf*1, *trn*K–*rps*16, *atp*H–*atp*I, *ndh*F, and *ndh*F–*rpl*32 reproduced the full plastome topology. These results provide plastome-scale evidence refining the phylogenetic placement of *A. ficulneus* and identify validated loci for molecular marker development within *Abelmoschus*.

## 1 Introduction

Okra (*Abelmoschus esculentus* (L.) Moench, Malvaceae) is an economically important crop widely cultivated in tropical and subtropical regions for its nutritional and agricultural value (Elhefnawi et al., 2023; Kwok et al., 2025). Despite its importance and notable phenotypic variability, the genetic relationships within the genus *Abelmoschus* remain incompletely resolved, partly due to the complexity of its evolutionary history and limited resolution of available molecular data (Mishra et al., 2017; Mkhabela et al., 2022). The genus comprises approximately 15 species and, although historically included within *Hibiscus*, is now recognized as a distinct monophyletic lineage within Malvaceae (Dong et al., 2025a).

*Abelmoschus ficulneus* (L.) Wight & Arn., a widely distributed wild species within the genus, has long been considered relevant to the evolutionary origin and diversification of cultivated okra; however, its taxonomic position remains unclear (Yuan et al., 2015; Mishra et al., 2017; Abhilash et al., 2026). Previous molecular systematics studies based on nuclear ITS and chloroplast *rpl16* sequence data (Werner et al., 2016) revealed inconsistent phylogenetic signals, with ITS analyses suggesting a relatively distant placement of *A. ficulneus* from *A. esculentus*, whereas *rpl*16 data indicated a closer association with related taxa. In contrast, classical breeding studies evaluating crossability and gene pool relationships (Pal et al., 1952) have indicated closer biological affinity among selected taxa. This discrepancy highlights the limitations of marker-based approaches and underscores the need for plastome-scale data to reassess the evolutionary placement of *A. ficulneus* within *Abelmoschus*.

Chloroplast genomes provide a robust framework for plant evolutionary studies due to their conserved structure, uniparental inheritance, and relatively low recombination rates (Cauz-Santos, 2025; Dong et al., 2025b; Lin et al., 2025, 2026). In angiosperms, plastomes typically exhibit a conserved quadripartite structure composed of large and small single-copy regions separated by inverted repeats and have been widely applied to resolve phylogenetic relationships at different taxonomic levels (Wicke et al., 2011; Ruhlman and Jansen, 2021). Advances in next-generation sequencing have enabled the assembly of complete plastomes, offering substantially higher resolution than traditional marker-based approaches (Qu et al., 2023; Bernal-Gallardo and de Folter, 2024).

Within Malvaceae and *Abelmoschus*, plastome studies have revealed conserved genome organization alongside variation at inverted repeat boundaries, including gene duplication events associated with IR expansion (Dong et al., 2025a). Such structural variation, particularly involving genes located at IR/SC junctions (e.g., *rps*3, *rpl*2, *rps*19), provides valuable evolutionary signals and may contribute to lineage differentiation (Lin et al., 2026; Wu et al., 2026). Previous studies have generated plastome resources for several Abelmoschus species and established comparative and phylogenetic frameworks based on plastid genomes (Li et al., 2020), while large-scale plastid phylogenomic analyses have further improved resolution of deep angiosperm relationships (Li et al., 2021). However, despite the availability of plastome data for multiple species, taxon sampling within the genus remains incomplete, particularly for wild relatives such as *A. ficulneus*.

In this study, we assembled and annotated the complete chloroplast genomes of cultivated okra (*A. esculentus*) and a wild relative *A. ficulneus*, representing the first report of its complete chloroplast genome and conducted a comparative plastome analysis across other wild species within the genus (*A. moschatus* Medik., *A. manihot* (L.) Medik., and *A. sagittifolius* (Kurz) Merr.), with *Hibiscus rosa-sinensis* L. as an outgroup. Using whole-plastome data, we reassessed evolutionary relationships within *Abelmoschus*, with particular emphasis on the placement of *A. ficulneus* relative to cultivated okra. We further evaluated plastome structural variation, including IR boundary shifts and gene duplication events. Furthermore, by identifying plastome regions that recapitulate phylogenetic signals derived from plastid genomes, this study provides candidate molecular markers that may improve the resolution of species relationships and complement previous marker-based and breeding evidence within *Abelmoschus*.

## 2 Materials and methods

### 2.1 Plant material, DNA extraction, and sequencing

Seeds of *Abelmoschus esculentus* (L.) Moench (accession AE12) were obtained from an in-house breeding line developed by the Genetics Department, Faculty of Agriculture, Ain Shams University, Egypt. Seeds of *A. ficulneus* (L.) Wight & Arn. were purchased from a commercial supplier (Seedbypost, India) via the Etsy platform. The taxonomic identity of *A. ficulneus* was subsequently verified using the chloroplast *rpl*16 marker, which has been widely used in Abelmoschus systematics (Werner et al., 2016). The obtained sequence showed 99.7% identity to published *A. ficulneus rpl*16 sequences (GenBank accessions AF384560 and KP222388).

Seeds of both species were germinated under controlled conditions, and plants were cultivated in the experimental field (for *A. esculentus*) and under indoor conditions (for *A. ficulneus*) until sufficient leaf biomass was obtained for DNA extraction. Fresh young leaves were collected, immediately frozen in liquid nitrogen, and stored at −80 °C until processing.

Total genomic DNA was extracted using a modified cetyltrimethylammonium bromide (CTAB) protocol following (Murray and Thompson, 1980) with optimization according to (Li et al., 2013). DNA integrity was verified by 1% agarose gel electrophoresis, and DNA concentration was quantified using a Quantus™ Fluorometer (Promega, USA) with the dsDNA quantification system. Sequencing libraries were prepared using standard Illumina protocols, and paired end reads (2 × 150 bp) were generated on an Illumina NovaSeq 6000 platform (Novogene, Munich, Germany), targeting approximately 30× coverage of the chloroplast genome.

### 2.2 Chloroplast genome assembly and annotation

Raw sequencing reads were evaluated using FastQC (Andrews et al., 2010) to assess base quality, GC content, and sequence duplication levels. Adapter sequences and low-quality bases were removed using Trimmomatic (Bolger et al., 2014) with trimming of bases below a Phred score threshold of 20. High-quality reads were assembled using an iterative reference-guided mapping approach implemented in Geneious Prime (Kearse et al., 2012), following the single-contig strategy described by (Magdy et al., 2019). Reads were mapped under medium sensitivity, and contigs were iteratively refined through successive rounds of remapping until a complete circular chloroplast genome was obtained. Assembly completeness was confirmed by the identification of the large single-copy (LSC), small single-copy (SSC), and two inverted repeat (IR) regions, with validation based on uniform read coverage and symmetry of IR sequences using repeat finder plugin in Geneious Prime. Genome annotation was performed in Geneious Prime and manually curated to verify gene boundaries, coding sequences, intron positions, and start/stop codons. Circular genome maps were generated using OGDRAW (Greiner et al., 2019).

### 2.3 Repeat analysis and codon usage bias

Simple sequence repeats (SSRs) were identified using the Phobos plugin (http://www.rub.de/ecoevo/cm/cm_phobos.htm, accessed on 20 March 2026), integrated in Geneious Prime, considering repeat unit sizes ranging from 2 to 10 bp, including both perfect and imperfect repeats (Androsiuk et al., 2020). Protein-coding sequences (CDS) were extracted from annotated plastomes and analyzed using MEGA v12 (Kumar et al., 2024) to calculate relative synonymous codon usage (RSCU) values, where RSCU > 1 indicates overrepresented codons and RSCU < 1 indicates underrepresented codons.

### 2.4 IR boundary analysis

The boundaries between the large single-copy (LSC), small single-copy (SSC), and inverted repeat regions were examined across all plastomes. Four junctions (JLB, JSB, JSA, and JLA) were defined and compared to detect IR expansion and contraction events. Visualization and comparative analysis of IR boundaries were conducted using IRscope (Amiryousefi et al., 2018), enabling precise identification of gene positioning at IR/SC junctions.

### 2.5 Comparative plastome analysis and identification of candidate markers

The comparative dataset comprised complete chloroplast genomes of *Abelmoschus esculentus* (AE12; this study), *A. ficulneus* (AF01; this study), and publicly available plastomes retrieved from GenBank, including *Hibiscus rosa-sinensis* (NC_042239) as an outgroup, *A. esculentus* (OL348389, ON641340, OQ947765), *A. moschatus* (MT890968), *A. manihot* (MT898000), and *A. sagittifolius* (MT898001).

The chloroplast genome sequences were aligned using MAFFT (Katoh and Standley, 2013). One copy of the inverted repeat (IR) region was removed prior to alignment to avoid redundancy. The alignment was manually inspected in Geneious Prime. Genetic diversity parameters, including the number of segregating sites (S), haplotype diversity (Hd), and nucleotide diversity (π), were calculated using DnaSP v6.12.03 (Rozas et al., 2017). Regions exhibiting elevated polymorphism were selected and evaluated for their ability to reproduce the phylogenetic topology inferred from complete plastome sequences. Loci, showing consistent clustering patterns were considered candidate markers for subsequent phylogenetic and taxonomic applications.

### 2.6 Phylogenetic analysis

Phylogenetic relationships were inferred using a maximum-likelihood (ML) approach implemented in FastTree v2 (Price et al., 2010) under the General Time Reversible (GTR) substitution model as implemented in Geneious Prime. Branch support values were estimated using the Shimodaira–Hasegawa (SH-like) approximate likelihood ratio test, and *Hibiscus rosa-sinensis* L. was used as an outgroup.

### 2.7 Selection pressure analysis (dN/dS)

Protein-coding sequences were aligned at the codon level using MAFFT (Katoh and Standley, 2013). Synonymous (dS) and nonsynonymous (dN) substitution rates were estimated in MEGA v12 (Kumar et al., 2024) using the Nei–Gojobori method with Jukes–Cantor correction. The ratio of nonsynonymous to synonymous substitutions (ω = dN/dS) was calculated for each gene to assess selective pressure.

## 3 Results

### 3.1 Organelle genome characteristics

**Figure 1:**
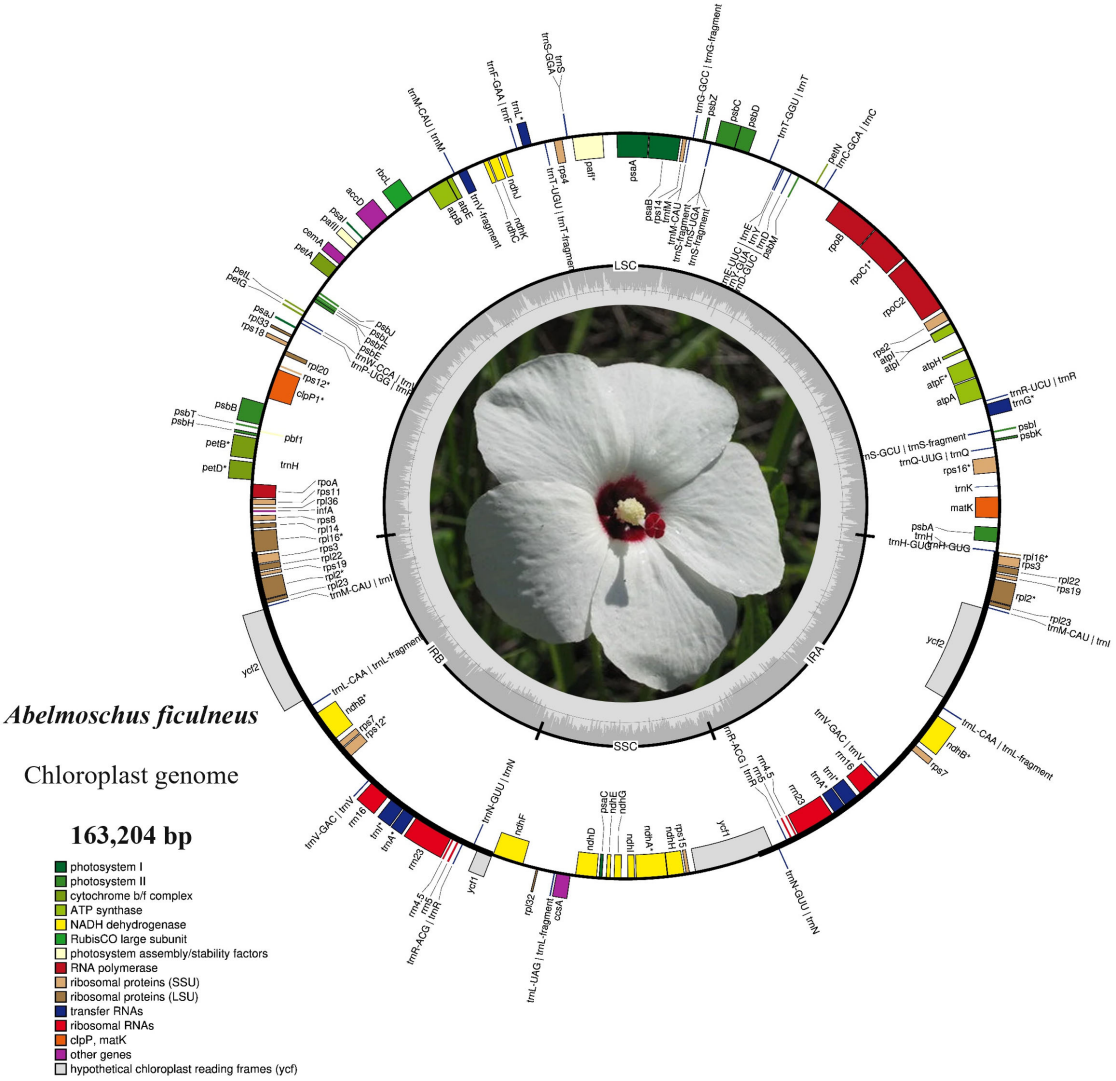
The complete chloroplast genome of *Abelmoschus ficulneus* species with total lengths of 163, 204 bp. The innermost circle illustrates the guanine–cytosine (GC) content and delineates the major chloroplast regions: the large single-copy (LSC), small single-copy (SSC), and inverted repeats (IRa and IRb). The outermost circle displays the full chloroplast sequence, with gene annotations positioned outside the circle for forward-strand genes and inside the circle for reverse-strand genes.

Comparative plastome analysis of *Abelmoschus* species, including the newly assembled *A. esculentus* (AE12) and the first complete chloroplast genome of *A. ficulneus* (AF01), revealed high structural conservation across all analyzed genomes. Among *Abelmoschus* species, plastome size ranged from 163, 119 bp to 163, 503 bp, and all genomes exhibited the conserved quadripartite structure (LSC, SSC, and two IRs) (**Figure 1**). The LSC, SSC, and IR regions varied within narrow ranges of 87, 998–88, 314 bp, 18, 815–19, 229 bp, and 28, 005–28, 162 bp, respectively. Size variation was limited and associated with minor boundary shifts, with no structural rearrangements detected.

The overall GC content was approximately 36.7% and % and remained highly conserved across all species. Nucleotide composition was similar among plastomes, with A, T, G, and C representing approximately 31.2%, 32.0%, 18.0%, and 18.7%, respectively (**Table 1**).

**Table 1:** The basic characteristics of *Abelmoschus* species and *Hibiscus rosa-sinensis* chloroplast genomes (LSC, large single-copy region; SSC, small single-copy region; IR, inverted repeat regions; GC%, guanine-cytosine percentage; length of the regions expressed in base pairs, bp).

*Species*	GC%	Length bp	LSC bp	SSC bp	IR bp	Genes (PCGs, tRNA, rRNA)
A. ficulneus (AF01)	36.7	163, 204	87, 998	18, 914	28, 146	133 (88, 37, 8)
A. esculentus (AE12)	36.7	163, 503	88, 256	19, 229	28, 009	133 (88, 37, 8)
A. esculentus (OL348389)	36.7	163, 121	88, 071	19, 032	28, 009	133 (88, 37, 8)
A. esculentus (ON641340)	36.7	163, 120	88, 071	19, 033	28, 008	133 (88, 37, 8)
A. esculentus (OQ947765)	36.7	163, 119	88, 061	19, 048	28, 005	133 (88, 37, 8)
A. moschatus (MT890968)	36.7	163, 430	88, 243	18, 931	28, 128	133 (88, 37, 8)
A. manihot (MT898000)	36.7	163, 428	88, 194	18, 934	28, 150	133 (88, 37, 8)
A.s sagittifolius (MT898001)	36.7	163, 453	88, 314	18, 815	28, 162	133 (88, 37, 8)
H. rosa-sinensis (NC_042239)	37.0	160, 951	89, 509	20, 246	25, 598	129 (84, 37, 8)

A total of 133 genes were annotated in *Abelmoschus* plastomes, including 88 protein-coding sequences (CDS), 37 tRNA genes, and 8 rRNA genes. In *H. rosa-sinensis*, the CDS number was slightly reduced (84 CDS) compared to the other analyzed species. Of the total genes, 113 were present as single-copy genes, whereas 20 genes were duplicated within the IR regions, including 9 CDS (*ndh*B, *rpl*2, *rpl*22, *rpl*23, *rps*3, *rps*7, *rps*12, *rps*19, *ycf*2), 7 tRNAs (*trn*N^GUU^, *trn*R^ACG^, *trn*A^UGC^, *trn*I^GAU^, *trn*V^GAC^, *trn*L^CAA^, *trn*I^CAU^), and 4 rRNAs (*rrn*5, *rrn*4.5, *rrn*16, *rrn*23). The gene *ycf*1 showed the greatest variation in length among species, and a total of 131 intergenic spacer regions were identified across the plastomes.

### 3.2 Tandem repeats and microsatellites

A total of 751 and 755 simple sequence repeats (SSRs) were identified in the chloroplast genomes of *A. esculentus* (AE12) and *A. ficulneus* (AF01), respectively. In both plastomes, hexanucleotide repeats were the most abundant class (187–188), followed by pentanucleotide (161) and tetranucleotide repeats (108–109), whereas trinucleotide (82) and dinucleotide repeats (69–71) were less frequent. Higher-order repeats (≥7 bp) occurred at lower frequencies in both genomes (**Table 2**).

**Table 2:** Repeated sequences in the chloroplast genomes of *Abelmoschus esculentus* (AE12) and *A. ficulneus* (AF01), showing repeat class, repeat abundances, and percentage abundance.

Chloroplast genome	Raw labels	IR	LSC	SSC	Total	~%
*Abelmoschus ficulneus* (AF01)	Dinucleotide repeat	26	41	4	71	9
	Trinucleotide repeat	32	47	3	82	11
	Tetranucleotide repeat	26	63	20	109	14
	Pentanucleotide repeat	43	99	19	161	21
	Hexanucleotide repeat	56	112	20	188	25
	Septanucleotide repeat	9	52	4	65	9
	Octanucleotide repeat	6	30	3	39	5
	Novanucleotide repeat	6	18	2	26	3
	Decanucleotide repeat	2	10	2	14	2
	Total	206	472	77	755	100
	Percentage %	27.2	62.5	10.1	100	ـ
Abelmoschus esculentus (AE12)	Dinucleotide repeat	24	40	5	69	9
	Trinucleotide repeat	32	45	5	82	11
	Tetranucleotide repeat	25	63	20	108	14
	Pentanucleotide repeat	43	101	17	161	21
	Hexanucleotide repeat	54	114	19	187	25
	Septanucleotide repeat	7	51	4	62	8
	Octanucleotide repeat	6	32	2	40	5
	Novanucleotide repeat	6	20	2	28	4
	Decanucleotide repeat	2	11	1	14	2
	Total	199	477	75	751	100
	Percentage %	26.4	63.5	9.9	100	ـ

SSRs were predominantly located in the large single-copy (LSC) region, accounting for 477 and 472 repeats in AE12 and AF01, respectively, followed by the inverted repeat (IR) regions (199 vs. 206) and the small single-copy (SSC) region (75 vs. 77).

**Figure 2:**
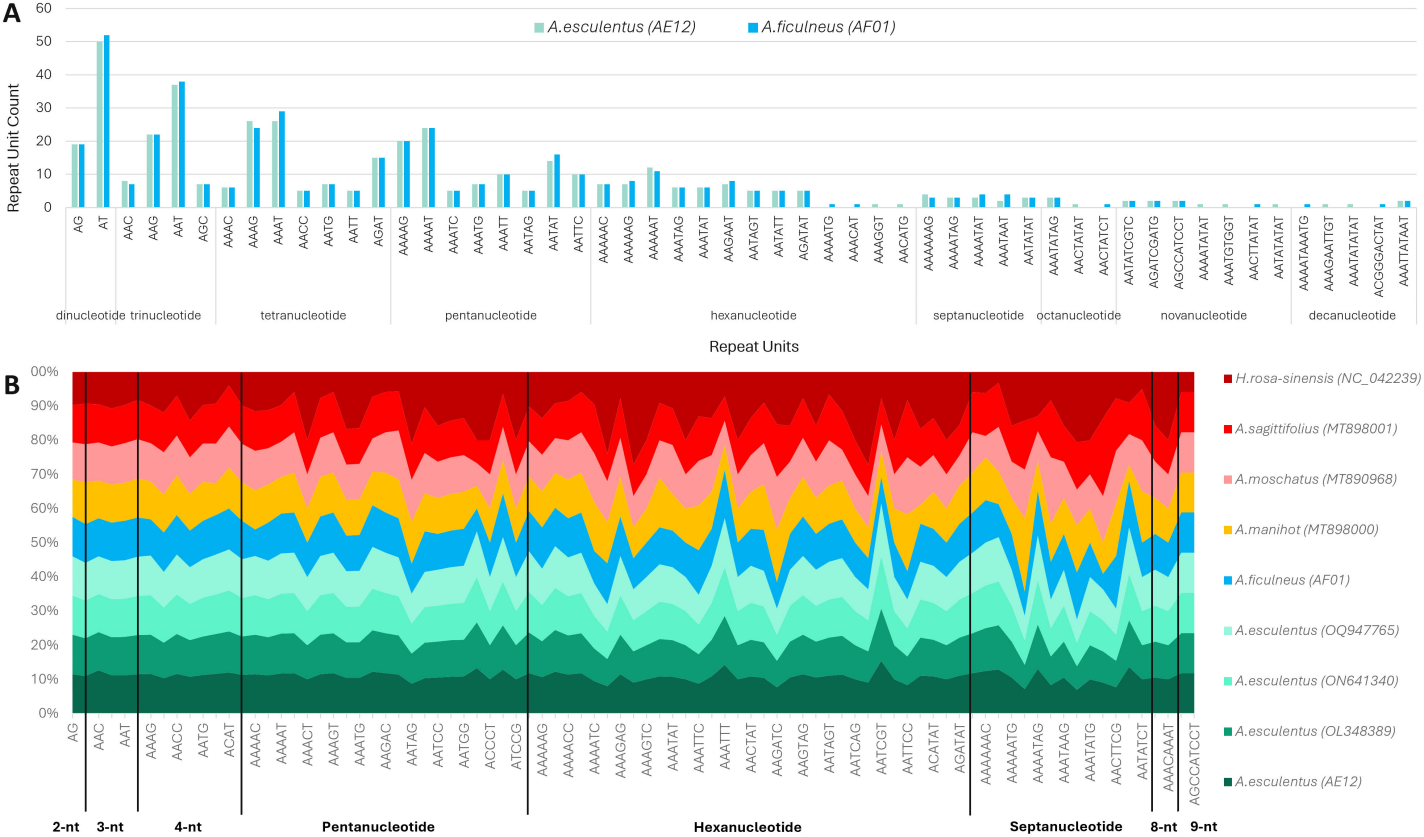
Distribution and composition of simple sequence repeats (SSRs) in *Abelmoschus* plastomes. **(A)** Motif-level distribution of SSRs in the chloroplast genomes of *A. esculentus* (AE12) and *A. ficulneus* (AF01), showing relative frequencies of repeat units. **(B)** Comparative distribution of SSR motifs across five *Abelmoschus* species and the outgroup *Hibiscus rosa-sinensis*, illustrating overall conservation and variation in motif composition among plastomes.

Motif composition was strongly AT-rich in both plastomes, with hexanucleotide and pentanucleotide repeats predominating. Motif-level distribution showed only minor variation between AE12 and AF01. Motif-level patterns showed only minor variation between the two genomes (**Figure 2A**). Across all Abelmoschus species, SSR abundance and composition were largely conserved, with total repeat numbers ranging from 745 to 758 per plastome. No clear species-specific clustering or enrichment of SSR motifs was observed, and overall patterns remained consistent across the genus (**Figure 2B**).

### 3.3 Relative synonymous codon usage and codon bias

**Figure 3:**
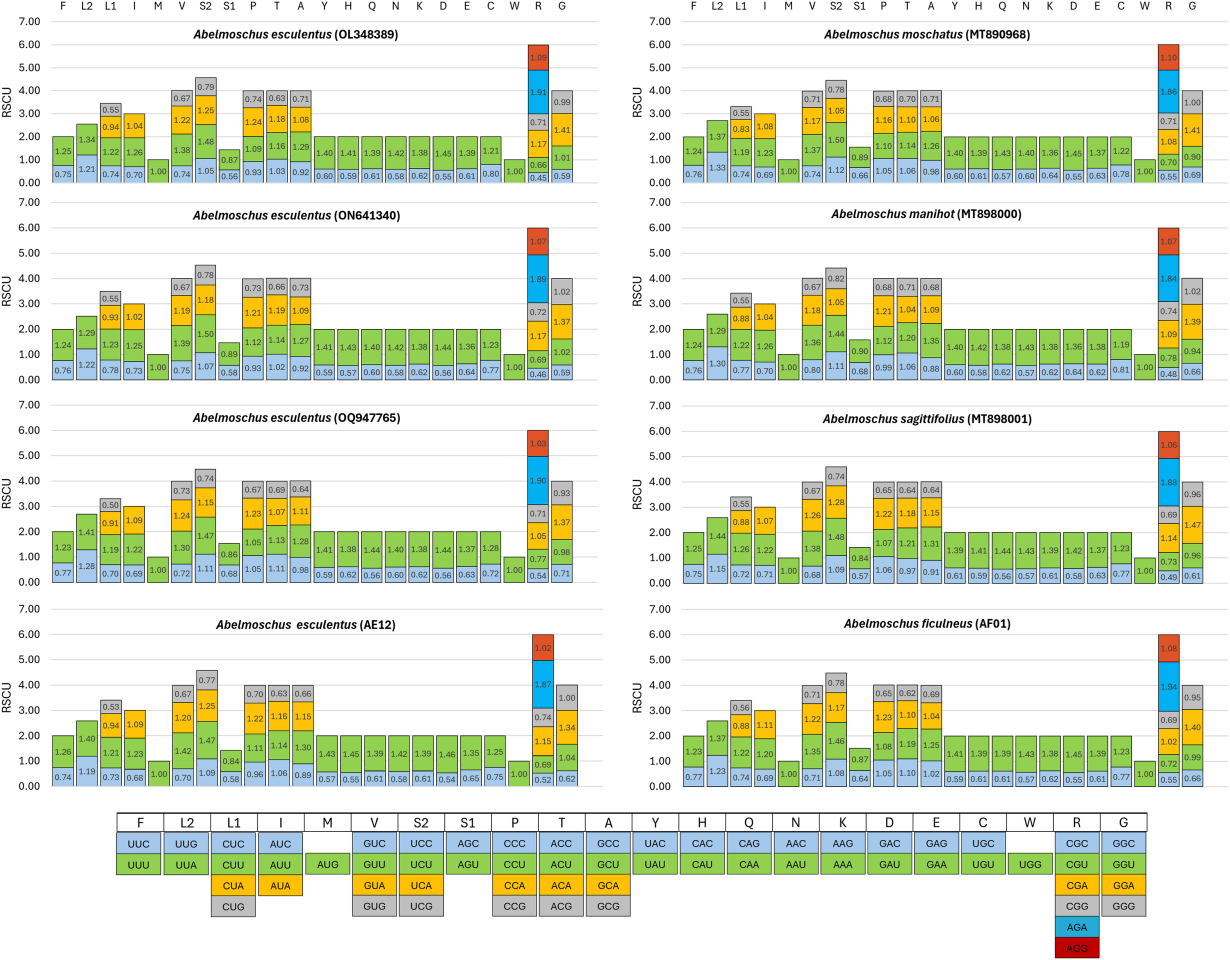
Relative synonymous codon usage (RSCU) patterns across five species of the genus *Abelmoschus* and *H. rosa-sinensis*. Stacked bar plots depict the RSCU values for each codon. The amino acids are represented using their one-letter abbreviations, and stop codons are indicated with *. Codons with RSCU values > 1 are overrepresented, while those with RSCU values < 1 are underrepresented.

Relative synonymous codon usage (RSCU) analysis was performed for 61 sense codons encoding 20 amino acids across six Malvaceae plastomes. Codon usage patterns were highly conserved across *Abelmoschus*, including *A. esculentus* (AE12) and *A. ficulneus* (AF01), with minimal interspecific variation (**Figure 3**).

A general bias toward A/U-ending codons at the third codon position was observed, consistent with the AT-rich composition of chloroplast genomes. Among amino acids encoded by multiple synonymous codons, arginine (Arg, R), serine (Ser, S), and leucine (Leu, L) displayed the strongest codon usage bias, with AGA (Arg) and UCU (Ser) overrepresented and CGC (Arg) and AGC (Ser) underrepresented. Similarly, UUA was generally preferred among leucine codons, whereas CUG showed consistently low usage. Amino acids with four synonymous codons, including threonine (Thr, T), alanine (Ala, A), and valine (Val, V), exhibited moderate and stable codon preferences, while those with two or three synonymous codons showed weaker but consistent bias toward A/U-ending codons. Stop codon usage was also conserved, with UAA as the predominant termination codon and UAG consistently underrepresented (**Figure 3**).

### 3.4 IR boundary contraction and expansion

**Figure 4:**
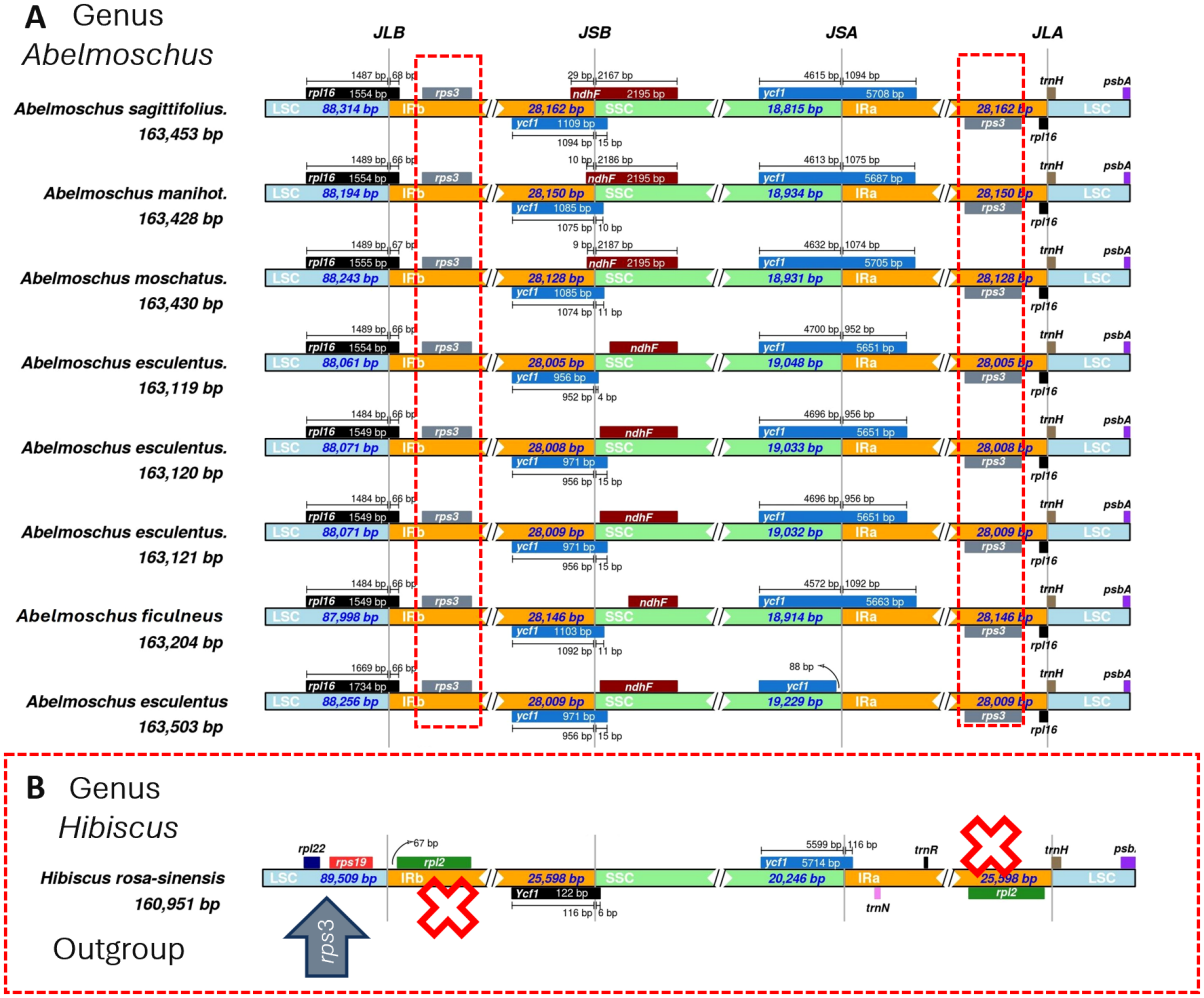
Comparison of chloroplast genome IR boundary regions in *Abelmoschus* and *Hibiscus*. **(A)** Structure and variation of LSC, IRb, SSC, and IRa junctions across *Abelmoschus* species, showing overall conservation with minor positional shifts of boundary genes. Red dashed boxes highlight the consistent localization of *rps*3 within IR regions. **(B)** Outgroup comparison with *Hibiscus rosa-sinensis*, illustrating a distinct IR boundary configuration in which *rpl*2 replaces *rps*3. Red crosses indicate the absence of *rps*3 at corresponding IR positions.

The inverted repeat (IR) regions were highly conserved across plastomes. Boundary junctions were defined as JLB (LSC/IRb), JSB (IRb/SSC), JSA (SSC/IRa), and JLA (IRa/LSC) (**Figure 4**). At the JLB junction, *rpl*16 was primarily located in the LSC region but extended partially into IRb in all *Abelmoschus* species, with a consistent overlap of approximately 66–68 bp. In contrast, the outgroup *Hibiscus rosa-sinensis* exhibited a distinct configuration, with *rpl*22 and *rps*19 confined to the LSC. Notably, the IRb region in *Abelmoschus* was characterized by the presence of *rps*3, whereas *rpl*2 occupied the corresponding region in *Hibiscus*, indicating a lineage-specific shift in IR gene composition (**Figure 4A**).

At the JSB junction, *ycf*1 was consistently positioned at the IRb/SSC boundary, extending slightly into the SSC region (4–15 bp), while *ndh*F remained predominantly within the SSC but overlapped IRb by 9–29 bp, showing minor interspecific variation.

At the JSA junction, *ycf*1 extended into the IRa region, with variation among species; notably, it was fully contained within the SSC in AE12 but extended into IRa in the remaining species, exceeding 100 bp in most cases and reaching 1094 bp in *A. sagittifolius*. At the JLA junction, *trn*H consistently defined the IRa/LSC boundary across all plastomes. In *Abelmoschus*, *rpl*16 was located near this junction, whereas *psb*A remained within LSC.

Importantly, *rps*3 was consistently located within the IRa region in *Abelmoschus*, in contrast to *rpl*2 in *Hibiscus*, reinforcing the distinct IR boundary configuration between the two genera. The consistent localization of *rps*3 within IR regions represents a distinctive genomic feature of *Abelmoschus*, differentiating it from the outgroup *Hibiscus* (**Figure 4B**).

### 3.5 DNA polymorphism and identification of hypervariable regions

DNA polymorphism was assessed across 271 chloroplast genomic regions, including coding sequences and intergenic spacers, using a dataset in which multiple *A. esculentus* accessions were collapsed into a single consensus sequence to enable direct interspecific comparison. Based on DnaSP analysis, mean genetic diversity across all regions was low, with an average nucleotide diversity (π) of 0.00116, mean haplotype diversity (Hd) of 0.147, and an average number of segregating sites (S) of 1.49 per region, consistent with the conserved nature of chloroplast genomes. Neutrality tests showed non-significant Tajima’s D values (mean = 0.231) across all regions.

Hypervariable regions were defined by the presence of five haplotypes (Hap = 5) and maximum haplotype diversity (Hd = 1), indicating complete sequence differentiation among taxa, regardless of the outgroup accession. A total of nine regions met these criteria, comprising both coding genes and intergenic spacers.

These regions included *trn*G (S = 8, π = 0.00471), *mat*K (S = 10, π = 0.00277), *ycf*1 (S = 25, π = 0.00215), *trn*K–*rps*16 (S = 17, π = 0.00999), *atp*H–*atp*I (S = 7, π = 0.00284), *psa*A–*paf*I (S = 5, π = 0.00242), *ndh*F (S = 15, π = 0.00319, Hd = 1), *ndh*F–*rpl*32 (S = 11, π = 0.00649), and *rpl*32–*trn*L (S = 7, π = 0.00322). These loci represent informative candidates for molecular marker development, capturing robust interspecific variation while maintaining alignment consistency.

### 3.6 Phylogenetic analysis

**Figure 5:**
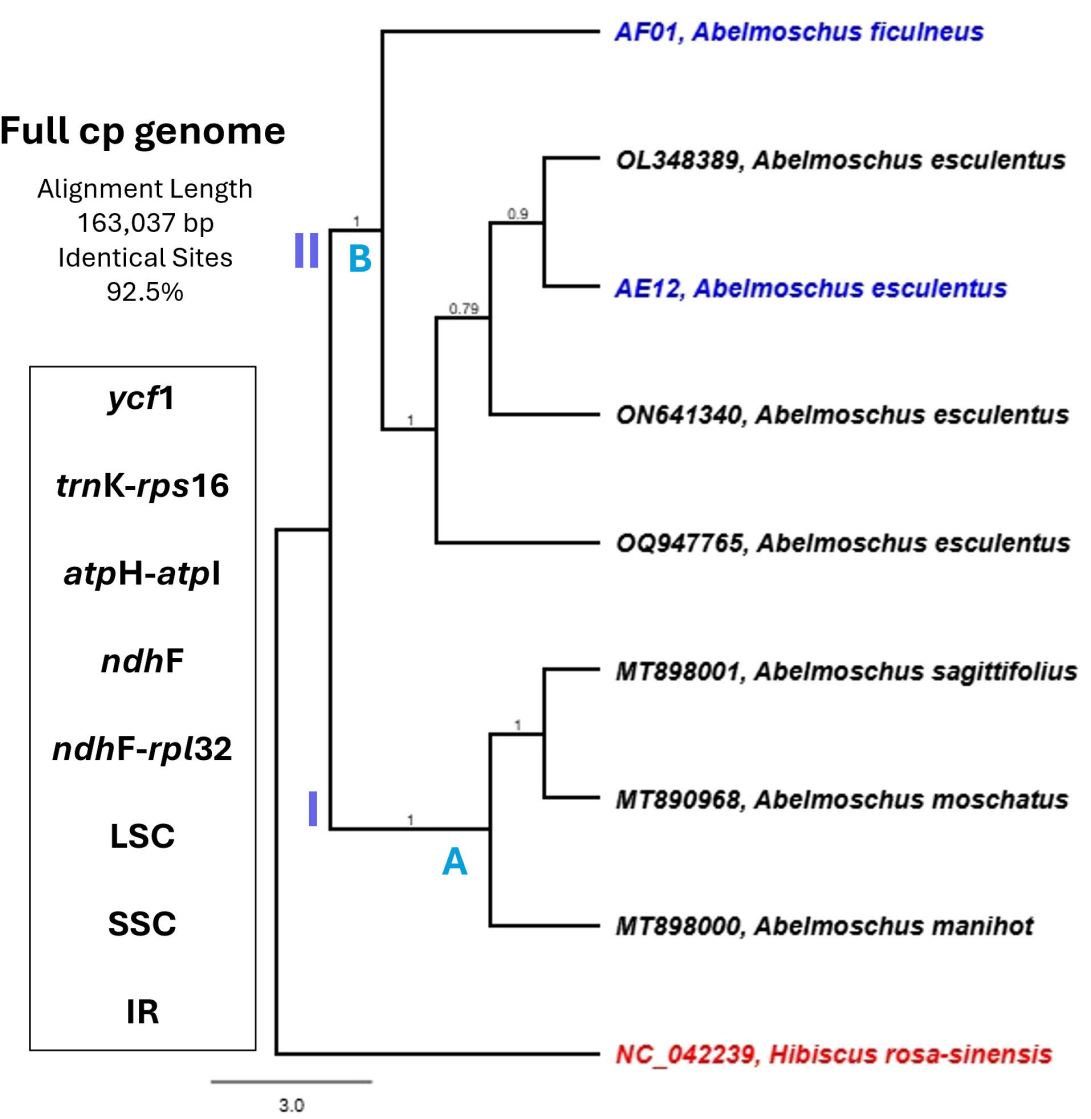
Maximum likelihood (ML) phylogenetic tree based on complete chloroplast genome sequences and selected genomic partitions in *Abelmoschus*, with *Hibiscus rosa-sinensis* as the outgroup. The full plastome alignment (163, 037 bp; 92.5% identical sites) resolved two major clades, with *A. ficulneus* clustering closely with *A. esculentus* accessions. Selected hypervariable loci (*ycf*1*, trn*K*–rps*16*, atp*H*–atp*I*, ndh*F, and *ndh*F*–rpl*32) and structural regions (LSC, SSC, IR) showed congruent phylogenetic signal with the complete plastome. Node values indicate SH-like support.

Phylogenetic relationships were reconstructed using complete plastome sequences and selected genomic partitions, including hypervariable loci and structural regions (LSC, SSC, and IR) (**Figure 5**). Maximum likelihood (ML) analysis, with *Hibiscus rosa-sinensis* as the outgroup, resolved all *Abelmoschus* species with strong SH-like support (≥ 0.99) across major nodes.

The full plastome phylogeny recovered two well-supported clades. Both clades were resolved as monophyletic. Clade I comprised *A. manihot*, *A. sagittifolius*, and *A. moschatus*, whereas Clade II included *A. ficulneus* and all *A. esculentus* accessions. Within Clade II, *A. ficulneus* formed a distinct lineage closely associated with cultivated *A. esculentus*.

Analysis of hypervariable loci demonstrated that *ycf*1, *trn*K–*rps*16, *atp*H–*atp*I, *ndh*F, and *ndh*F–*rpl*32, as well as the full LSC, SSC and IR regions retained strong phylogenetic signal and successfully reconstructed relationships consistent with the full plastome tree.

### 3.7 Nonsynonymous to synonymous substitution ratio

**Figure 6:**
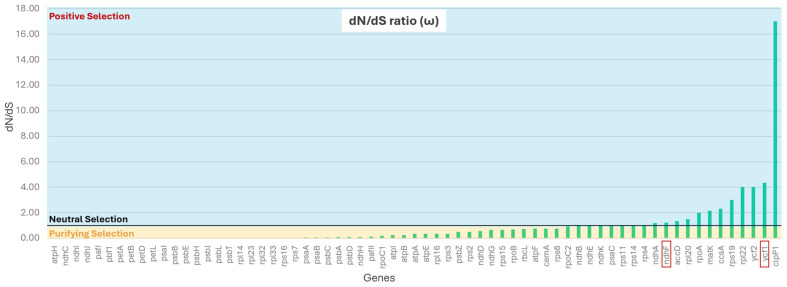
Distribution of nonsynonymous-to-synonymous substitution ratios (dN/dS; ω) across chloroplast protein-coding genes. Most genes exhibit ω < 1, consistent with purifying selection, whereas a smaller subset shows ω > 1. Genes with ω values approaching neutrality (ω ≈ 1) are also observed. The horizontal line indicates the neutrality threshold (ω = 1). The genes *ycf*1 and *ndh*F are highlighted due to their prominence among variable loci.

Selective pressure on chloroplast protein-coding genes was assessed using the nonsynonymous to synonymous substitution ratio (dN/dS; ω). Most genes exhibited ω < 1, indicating that purifying selection is the predominant evolutionary force maintaining chloroplast gene function (**Figure 6**).

A limited number of genes showed ω > 1, suggesting signatures of positive selection. Among these, *clp*P, *ycf*1, *ycf*2, and *rpl*22 displayed the highest ω values, indicating strong evidence of adaptive evolution. Additional genes, including *rps*19, *ccs*A, *mat*K, and *rpo*A, also exhibited elevated ω values, consistent with potential functional divergence. Several genes, such as *ndh*F, *ndh*A, *acc*D, and *rpl*20, showed ω values close to neutrality (ω ≈ 1), suggesting relaxed selective constraints or episodic selection.

## 4 Discussion

A typical angiosperm chloroplast genome consists of two IR regions, an LSC region, and an SSC region. This quadripartite organization is highly conserved across flowering plants and reflects a stable and evolutionarily constrained plastid genome structure (Li et al., 2025). Comparative plastome analysis of *Abelmoschus* revealed a high degree of structural conservation, with only minor variation in genome size and GC content. Gene order and gene content were consistent with previous reports for Malvaceae plastomes (Dong et al., 2025a; Maurya et al., 2025; Abdullah et al., 2026) indicating strong evolutionary constraints on chloroplast genomes. Notably, re-annotation identified an intact chloroplast-encoded *inf*A gene that was not reported in the previously published *Abelmoschus* plastomes included in the present comparative analysis (Dong et al., 2025a). The *infA* gene, encoding a translation initiation factor, has been independently lost multiple times during the evolution of land plants (Millen et al., 2001), and its presence and functional status vary among Malvaceae species, where it may occur in either functional or non-functional forms (Li et al., 2020). However, given the well-documented evolutionary history of *infA* involving frequent loss or transfer to the nuclear genome in angiosperms, its functional status remains uncertain (Millen et al., 2001). Plastome sizes fell within the previously reported range for *Abelmoschus*, further confirming genome stability. These findings collectively support strong structural conservation of the plastome in Abelmoschus, with only minor lineage-specific variation.

SSRs, also known as microsatellites, are highly informative molecular markers widely used in plant genetic improvement due to their high mutation rates and ubiquitous distribution across genomes, which provide strong discriminatory power for species identification, phylogenetic reconstruction, and the analysis of evolutionary processes (Saeed et al., 2016; Zhong et al., 2022). Microsatellite profiling revealed a predominance of AT-rich SSR motifs, with hexanucleotide repeats representing the most abundant class. This pattern is consistent with the compositional bias of plastid genomes and previous observations in Malvaceae (Fredrick Onyango et al., 2026; Yan et al., 2026). Despite minor variation in repeat frequencies, SSR distribution was highly conserved across species, indicating shared genomic features within *Abelmoschus*. These results suggest that while SSRs may have limited resolution for deep phylogenetic inference, they remain useful for population-level studies and marker development, as previously reported for related taxa (Ergashov et al., 2026).

Codon usage analysis revealed a strong bias toward A/U-ending codons, consistent with the AT-rich nature of plastid genomes and widely observed across angiosperms (Liu et al., 2024; Shen et al., 2024; Gong et al., 2025; Zou et al., 2025). The highly conserved codon usage patterns among Abelmoschus species indicate that synonymous codon evolution is predominantly governed by mutational bias associated with nucleotide composition, rather than by strong lineage-specific selection. This pattern is consistent with the conserved evolutionary dynamics of plastid genomes, in which efficient DNA repair, homologous recombination, and genome maintenance mechanisms limit mutation accumulation and help preserve genome integrity (Sloan and Taylor, 2012; Barnard-Kubow et al., 2014; Smith, 2015; Wang et al., 2024). Although translational selection may influence codon preference in a limited subset of highly expressed genes by enhancing translational efficiency and accuracy, comparative studies suggest that its contribution to genome-wide codon usage is generally secondary to mutation pressure in angiosperm plastomes (Suzuki and Morton, 2016). Minor interspecific variation was observed but did not alter the overall codon usage profile, indicating that codon usage has remained evolutionarily conserved throughout the diversification of the genus.

Expansion and contraction of IR boundaries are major drivers of plastome size variation and contribute to interspecific differences in genome structure and gene content (Goulding et al., 1996; Wang et al., 2022; Cauz-Santos, 2025). Minor shifts were observed at the IR/SC junctions among Abelmoschus plastomes. A notable feature distinguishing *Abelmoschus* from the outgroup *Hibiscus rosa-sinensis* was the consistent localization of *rps*3 within IR regions, in contrast to *rpl*2 in *Hibiscus*. This shift represents a genus-specific structural characteristic, consistent with lineage-specific IR evolution reported in Malvaceae (Kwon et al., 2023). Such boundary shifts, although subtle, provide useful phylogenetic signals and contribute to plastome diversification.

A key outcome of this study is the identification of nine hypervariable regions that exhibit complete haplotype differentiation across taxa. Although chloroplast genomes are generally conserved, they still contain mutational hotspots widely used for species identification and DNA barcoding, including *rbc*L, *mat*K, *ndh*F, and *psb*A-*trn*H, as also reported in Malvaceae (Zhong et al., 2024). Among these loci, *rbc*L and *mat*K are widely recognized as core plant DNA barcode markers (Hollingsworth et al., 2009). These regions include both coding genes and intergenic spacers, indicating that informative variation is distributed across functional categories. Several of the detected hotspots, such as *trn*K–*rps*16, *atp*H–*atp*I, *ycf*1, and *ndh*F, are consistent with previously identified variable regions in Malvaceae plastomes (Alzahrani, 2021). Compared with standard plastid markers, these loci are potential candidates for improved resolution in species discrimination and phylogenetic inference in *Abelmoschus*, although their performance requires further validation across broader sampling.

Phylogenetic reconstruction based on complete plastomes resolved two well-supported clades, with *A. ficulneus* consistently clustering with *A. esculentus*. This result contrasts with earlier marker-based analyses, particularly those based on ITS and limited chloroplast loci, which suggested a more distant placement of *A. ficulneus* (Werner et al., 2016). The present plastome-scale analysis provides stronger and more consistent support for a close evolutionary relationship, highlighting the limitations of single-locus approaches and emphasizing the importance of genome-wide data. Moreover, several hypervariable loci (*ycf*1, *trn*K*–rps*16, *atp*H*–atp*I, *ndh*F, and *ndh*F*–rpl*32) successfully recapitulated the full plastome topology, demonstrating their utility as cost-effective phylogenetic markers for phylogenetic reconstruction within Abelmoschus. Nevertheless, their robustness and transferability should be further assessed using broader taxonomic sampling and independent datasets.

Analysis of selective pressure indicated that most chloroplast genes are under purifying selection, reflecting functional conservation typical of plastid genomes and commonly reported across comparative plastome studies in angiosperms (Wang et al., 2026b, 2026a). A limited number of genes, including *clpP*, *ycf1*, *ycf2*, and *rpl22*, showed elevated dN/dS ratios, which may reflect relaxed purifying selection, lineage-specific divergence, recombination, or stochastic effects associated with limited taxon sampling and short evolutionary distances, rather than definitive evidence of adaptive evolution. These patterns suggest moderate variation in selective constraints among genes (Wertheim et al., 2015). Because the evolutionary dynamics of dN/dS are inherently time-dependent, elevated ω values are best interpreted in conjunction with gene function, sequence variability, and broader evolutionary context, particularly among closely related lineages (Mugal et al., 2014). Among these genes, *ycf1* exhibited both high variability and elevated ω values, reinforcing its utility as an informative molecular marker for phylogenetic inference and comparative plastome analyses within *Abelmoschus* and related Malvaceae lineages (Li et al., 2020; Wang et al., 2021). In contrast, *ndhF*, although highly variable, displayed ω values close to neutrality, indicating that its sequence divergence has accumulated while maintaining overall functional conservation. Collectively, these findings indicate that plastome evolution in *Abelmoschus* is dominated by purifying selection (Li et al., 2020), while a small subset of genes exhibits moderate variation in selective constraints that may be associated with lineage-specific divergence.

## 5 Conclusion

Plastome-scale analysis provides robust resolution of evolutionary relationships within *Abelmoschus*. The consistent placement of *A. ficulneus* alongside cultivated *A. esculentus* supports a close evolutionary affinity, refining previous interpretations based on limited molecular markers. The identification of a restricted set of hypervariable loci capable of reproducing full plastome phylogenetic structure demonstrates that high-resolution inference can be achieved without complete genome data, offering practical tools for future taxonomic and genetic studies. In addition, the conserved structural organization of plastomes, coupled with lineage-specific IR boundary features, reinforces the stability of chloroplast genomes while highlighting subtle genomic signatures of divergence within the genus. These findings establish a refined phylogenetic framework for *Abelmoschus* and provide a targeted set of loci for downstream applications in systematics and molecular characterization.

## Data Availability

The complete chloroplast genomes of Abelmoschus ficulneus (AF01) and Abelmoschus esculentus (AE12) for this study can be found in figshare: https://doi.org/10.6084/m9.figshare.32635695.
